# Risk Factors Associated with Cognitive Decline after Cardiac Surgery: A Systematic Review

**DOI:** 10.1155/2015/370612

**Published:** 2015-09-30

**Authors:** Nikil Patel, Jatinder S. Minhas, Emma M. L. Chung

**Affiliations:** ^1^Department of Cardiovascular Sciences, University of Leicester, Leicester LE2 7LX, UK; ^2^Leicester Cardiovascular Biomedical Research Unit, Glenfield Hospital, Leicester LE3 9QP, UK; ^3^University Hospitals of Leicester NHS Trust, Leicester LE1 5WW, UK; ^4^Department of Medical Physics, University Hospitals of Leicester NHS Trust, Leicester LE1 5WW, UK

## Abstract

Modern day cardiac surgery evolved upon the advent of cardiopulmonary bypass machines (CPB) in the 1950s. Following this development, cardiac surgery in recent years has improved significantly. Despite such advances and the introduction of new technologies, neurological sequelae after cardiac surgery still exist. Ischaemic stroke, delirium, and cognitive impairment cause significant morbidity and mortality and unfortunately remain common complications. Postoperative cognitive decline (POCD) is believed to be associated with the presence of new ischaemic lesions originating from emboli entering the cerebral circulation during surgery. Cardiopulmonary bypass was thought to be the reason of POCD, but randomised controlled trials comparing with off-pump surgery show contradictory results. Attention has now turned to the growing evidence that perioperative risk factors, as well as patient-related risk factors, play an important role in early and late POCD. Clearly, identifying the mechanism of POCD is challenging. The purpose of this systematic review is to discuss the literature that has investigated patient and perioperative risk factors to better understand the magnitude of the risk factors associated with POCD after cardiac surgery.

## 1. Introduction

Neurological complication after cardiac surgery is of a considerable concern and debate exists as to which perioperative factors may be responsible for this adverse injury. Significant advances in all aspects of intraoperative and postoperative care mean cardiac surgery is now safer than ever before [1]. However, as the complexity of surgical procedures increases and the population ages, neurological manifestations and adverse cognitive outcomes are of concern. Cognitive decline limits the ability to complete activities of daily living [2] and increase the likelihood of dependence after discharge [3]. It is therefore paramount to determine the aetiology and extent of brain injury. Such complications vary from subtle cognitive impairment to catastrophic stroke events. The complexity of the brain is demonstrated by small lesions potentially causing significant loss of function, with larger lesions on occasion causing asymptomatic outcomes.

Over time, the demographic characteristics of patients undergoing cardiac surgery have shifted to include a higher proportion of elderly patients, undergoing increasingly complex procedures. The average age of cardiac surgery patients has increased from ~64 years in 2001 to ~67 years in 2010. The number of patients with neurological disease prior to surgery has nearly doubled from 1.4% in 2001 to ~2.8% in 2010. Cardiac surgery procedures have also become more complex, with the number of patients undergoing isolated coronary artery bypass graft (CABG) decreasing by almost 20% from 2001 to 2010. Despite higher patient risk profiles, the mortality rate has fallen slightly from 4.0% in 2001/2002 to 3.1% in 2010/2011 (National Cardiac Surgery Audit, UCL, 2012).

Routine clinical examination covers crucial neurological abnormalities such as ataxia, visual defects, paresis, and hypaesthesia [4]. It also includes focal neuropsychological deficits such as apraxia, dyscalculia, and aphasia. However, more global cerebral dysfunction, such as neuropsychological decline, mood, and memory disturbances, personality changes, and decline in psychomotor speed are commonly missed because they require more explicit examination using specialised neuropsychological tests [5]. Postoperative cognitive decline (POCD) broadly refers to difficulties associated with memory and general information processing after surgery. At present POCD is not documented in the International Classification of Diseases and is not listed as a diagnosis.

## 2. Methods

### 2.1. Data Sources

A systematic literature search was conducted from searching articles from PubMed and EMBASE. Search terms were created by combining the following medical subject headings (MeSH terms): “Coronary Artery Bypass” OR “Coronary Artery Bypass, Off-Pump” OR “Valve Surgery” OR “Thoracic Surgery” OR “Cardiac Surgical Procedures” AND “Cognitive Therapy” OR “Cognition Disorders” OR “Cognition” OR “Neuropsychology” OR “Neuropsychological Tests” OR “Mild Cognitive Impairment.”

### 2.2. Study Selection

All studies published in English between June 1967 and August 2014 and featuring adult human subjects were eligible for review. Abstracts were excluded if they involved paediatric surgery, operations other than cardiac surgery, or no measurement of cognitive function. Case reports and studies of cardiac procedures such as angioplasty, angiography, valvuloplasty, and Transcatheter Aortic Valve Implantation (TAVI) were also excluded. Studies generating multiple publications from the same cohort were reported only once.

### 2.3. Quality Assessment

Abstracts involving both cardiac surgery and cognitive function were independently reviewed by two investigators (Nikil Patel and Emma M. L. Chung) and studies of adult cardiac surgery patients that assessed both before and after operative cognitive function were identified for full paper review. Where there was disagreement among investigators the full text was reviewed. Additionally, the reference lists of selected articles were evaluated for any additional articles of interest.

### 2.4. Analysis

Articles short-listed for full manuscript review were summarised in an Excel spreadsheet listing the study design (observational, RCT, etc.), number of patients, type(s) of surgery, outcome measures, and time point of neurocognitive assessment. Studies that included assessment of anxiety and depression were also recorded, as these conditions can impact the outcome of cognitive assessments. There was insufficient homogeneity between studies to allow a quantitative, meta-analytic approach of region of interest studies. Therefore, a critical, systematic review was undertaken.

## 3. Results

A total of 638 abstracts were systematically identified using our search criteria of which 426 papers were suitable for full review. Of these, 296 were observational studies and 130 were RCTs. Although over 420 original research articles were identified as having investigated cognitive decline following cardiac surgery, we found little consensus on the incidence, severity, and time course of symptoms. Differing methodologies used between studies made it difficult to directly compare study findings through systematic meta-analysis.

### 3.1. Time of Postoperative Testing and Cognitive Decline

**Figure 1:**
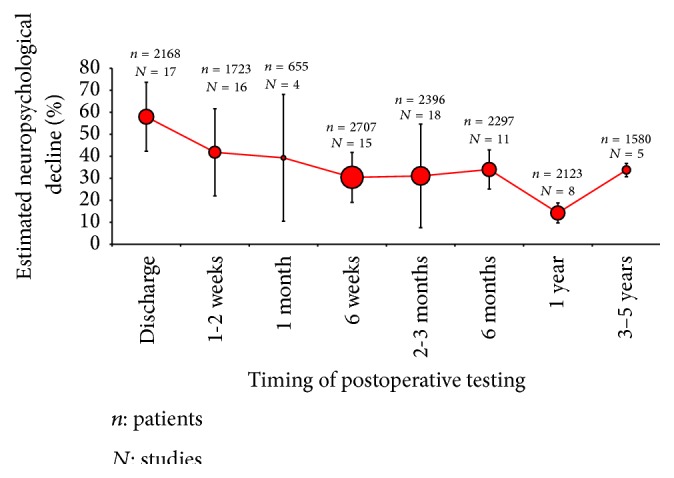
Studies attempting to quantify neuropsychological decline at various time points. The weighted mean and standard deviation (number of patients and % decline) are plotted by combining data from a total of 15649 patients and 94 studies; discharge (17 studies), 1-2 weeks (16 studies), 1 month (4 studies), 6 weeks (15 studies), 2-3 months (18 studies), 6 months (11 studies), 1 year (8 studies), and 3–5 years (5 studies).

Most studies evaluating cognitive decline focus on changes in executive function, learning language, visual spatial skills, attention, and memory [6]. However, neuropsychological tests vary considerably between studies and also appear to depend on the timing of neurocognitive assessment. By narrowing the search to empirical research articles that studied postoperative neuropsychological assessment as a primary outcome, the number of publications was reduced to 137 articles. Thirty-three of these articles were excluded because the total percentage of patients who declined in cognitive tests was unclear. Four articles had published the same data twice and full-texts were unavailable for 6 articles. A total of 94 studies were identified to establish the distribution of cognitive decline over several time points. Grouping studies where assessments were performed at similar time points and plotting the proportion of patients estimated to be affected by cognitive decline suggest that 40–60% of patients experience cognitive decline when tested within 2 weeks of surgery, falling to 30–40% after 8–10 weeks, recovering to 10–20% at 1 year, with proportion of patients experiencing cognitive decline increasing again at 3–5 years, Figure 1.

Large variations in the estimated incidence of postoperative cognitive decline are observed, even after grouping studies where tests were performed at similar time points, Figure 1. Heterogeneity in assessment methods, patient demographics, and study design may be responsible for these variations.

### 3.2. Perioperative Risk Factors and Cognitive Decline

Further, we investigated perioperative risk factors associated with cognitive decline. Potential mechanisms implicated in the pathogenesis of cognitive decline investigated in previous research resulted in a total of 92 articles (see PRISMA chart in Supplementary Material available online at http://dx.doi.org/10.1155/2015/370612): anaesthesia: 15 studies; blood pressure: 5 studies; cerebral autoregulation: 4 studies; inflammatory responses: 26 studies; neuroprotective agents: 17 studies; hypothermia and rewarming: 19 studies and 6 studies, respectively.

### 3.3. Anaesthesia

Sedative and anaesthetic agents with* N-methyl-d-aspartate* receptor antagonist and *γ-aminobutyric acid* mediated properties can temporarily change the neurotransmission of the brain by interacting at a cellular level to achieve deep sedation during surgery [7]. Since it would be unethical to perform cardiac surgery without the use of anaesthetic agents, the impact of anaesthesia on cognition is difficult to study. Fifteen studies have investigated whether choice of anaesthesia impacts neurocognitive outcome after cardiac surgery. Of these, 8 were randomised controlled trials (RCTs), comparing 7 different types of anaesthetic agent. Studies showing an improvement, decline, and no difference in postoperative outcome are summarised in Table 1.

This research suggests that choice of anaesthetic has potential to affect cognition, particularly when tests are performed soon after surgery. However, in the majority of larger studies, the choice of anaesthetic had no impact on cognitive outcome.

### 3.4. Blood Pressure

A number of studies have investigated the association between low blood pressure during cardiac surgery and cognitive decline. Although normal blood pressure in conscious patients is approximately 120/80* *mmHg, it is common for the blood pressure to be much lower during surgery. As the brain has a lower metabolic demand during anaesthesia, this is not thought to adversely affect tissue perfusion; however, low blood pressure may impair embolus clearance and affect the efficiency of cerebral autoregulation. A total of 5 studies have used neuropsychological tests to investigate whether mean arterial blood pressure had any impact on postoperative cognitive outcome, Table 2.

In the study by Gold et al., a higher mean arterial pressure (80–110 mmHg) during CPB appeared to be associated with a lower stroke rate (2.4%) compared to a low mean arterial pressure between 45 and 60 mmHg (7.2%), *p* = 0.026. However, at 6-month follow-up the proportion of patients with neuropsychological declines (11% and 12%, resp.) were comparable [8]. In another study, Siepe et al. showed greater proportion of patients with cognitive decline two days following CABG in patients with mean arterial pressure in the range 60–70 mmHg compared to 80–90 mmHg; however cerebral oxygen saturation was similar in both groups [9]. The largest RCT by Charlson et al. found no difference in cognition between a “custom” group (average BP: 79 mmHg) and High BP group (average BP: 89 mmHg); however, the average difference in BP between groups was only 10 mmHg, which may not be a clinically significant difference [10]. Overall, studies appear to support the idea that maintenance of a sufficiently high mean arterial pressure during cardiac surgery is important for safeguarding perfusion to the brain.

### 3.5. Cerebral Autoregulation

Some researchers have proposed that it is not mean arterial pressure (MAP)* per se* that contributes to cognitive decline, but the capacity of the brain's blood flow regulation mechanisms to respond appropriately to blood pressure variations and changes in oxygen saturation. A number of studies have investigated cerebral autoregulation (CA) in response to blood pressure changes during cardiac surgery and found that a significant proportion of patients struggle to autoregulate their cerebral blood supplies intraoperatively [11]. However, only 4 studies have specifically investigated CA during cardiac surgery in conjunction with pre- and postoperative neuropsychological assessment, Table 3.

All four studies determined pressure-flow and metabolic-flow cerebral autoregulation during cardiopulmonary bypass using the ^133^Xe clearance cerebral blood flow method. Two studies in Table 3 by the same author (Patel et al.) support the theory that impaired cerebral autoregulation is associated with a decline in postoperative outcome at 6 weeks, whereas two studies showed no association. The largest study by Newman et al. investigated CA in 215 patients and concluded that neuropsychological dysfunction at discharge was not explained by impaired CA; however increased oxygen extraction (measured using a thermodilution pulmonary artery catheter) was observed to be associated with a decline in some cognitive tests. They interpreted this suggesting that an imbalance in cerebral tissue oxygen supply may contribute to POCD [12]. In a recent trial it has also been proposed that some anaesthetic agents suppress autoregulatory responses more than others [13]. As far as we are aware, no studies have yet looked at the relationship between CA and POCD beyond 6 weeks.

### 3.6. Inflammatory Responses

All types of surgery have the risk of developing systemic inflammation; however, in cardiac surgery using CPB the blood is exposed to foreign surfaces which have potential to stimulate proinflammatory responses. Inflammation causes endothelial dysfunction, which can lead to leakage between the blood-brain barrier and tissue oedema [14]. It has been shown that cytokines (e.g., TNF-alpha, interleukin-1, and interleukin-6) have been linked to neuropathology [15, 16]. These elementary changes are hypothesised to affect the brain regardless of microembolic load received during surgery [17, 18] and potentially provide an explanation for early cognitive decline [19].

Cardiopulmonary bypass components that come into contact with the blood can be coated with biocompatible materials such as poly-2-methoxyethylacrylate, heparin, trillium, and synthetic proteins. These coatings aim to reduce inflammatory responses triggered during CPB. Heparin-coated circuits, in particular, have undergone considerable investigation in previous research. A total of 26 studies (including 7 RCTs) have used neuropsychological tests to investigate whether there is a strong association between inflammation and cognitive decline, Table 4.

All studies that have randomised patients to receive a heparin-coated CPB system found neuropsychological outcome was better in patients receiving the heparin-coated circuit [19–22]. In studies investigating inflammatory responses, a consensus panel has concluded that “*the use of surface-modified circuits might be effective at attenuating the systemic inflammatory response to CPB and improving outcome*” [23]. Many markers associated with susceptibility to brain ischaemia such as* S-*100 beta and neuron-specific enolase (NSE) have been suggested to be associated with an increased risk of cognitive decline [24–26]. Inflammation may also play an important role in our understanding of long-term cognitive function. Biomarkers for inflammation tend to be higher in patients with chronic cardiovascular disease [25]. Overall, the role of inflammation in the pathogenesis of cognitive decline appears to warrant further investigation [27].

### 3.7. Neuroprotective Agents

A number of neuroprotective agents have been investigated to assess whether these could be administered to help preserve neurocognitive function. The results of 17 studies investigating whether neuroprotective agents reduce the incidence of POCD are summarised in Table 5.

One of the most commonly used neuroprotective agents is Lidocaine, which featured in 4 of the 17 studies. Lidocaine is thought to inhibit inflammatory responses during cardiac surgery by modulation of inflammatory mediators, reduction in cerebral metabolism, and deceleration of ischaemic ion fluxes [28]. Two studies showed improved outcome with the use of the drug [29, 30], while two studies showed no difference [31, 32]. Currently, no trials have demonstrated a reproducible clinically significant benefit conferred by the use of any particular neuroprotective drug.

### 3.8. Hypothermia and Rewarming

The patient's temperature during cardiac surgery has long been thought to play a role in neurological outcome. Several studies have focused their trials on whether reducing the metabolic demand of the brain through hypothermia is neuroprotective. Based on our literature search, 41 studies investigating the effects of temperature were identified. Seventeen studies were excluded from the final result due to lack of clarity in neuropsychological assessments and outcomes. Results from a total of 19 studies investigating the effect of temperature on pre- and postoperative neuropsychological tests are summarised in Table 6.

Some studies suggest that hypothermia is more effective than normothermia in protecting the brain during surgery; however, other studies report no obvious difference between “mild hypothermia” and “normothermia” in terms of neuropsychological performance at discharge (49% and 45%, resp.) and at 3 months (4% and 8%, resp.) [33].

Some researchers have proposed that the brain could be susceptible to insult during rewarming from hypothermia, particularly if cerebral autoregulation mechanisms are unable to compensate for a sudden increase in metabolic activity associated with changes in temperature. Six studies have been conducted to examine the effect of rewarming rate on POCD, and all of these have shown a benefit in postoperative outcome associated with slower rewarming, Table 7.

## 4. Conclusion

Neuropsychological function is a soft outcome measure and has proved challenging to quantify postoperatively. Although neuropsychological tests theoretically provide a highly sensitive means of quantifying changes in cognition, differences in test batteries, timing of assessment, and criteria for defining neuropsychological decline generate considerable heterogeneity in the data, which limits our ability to compare the results of different studies. Depending on the timing of the neurocognitive tests and the definition used for determining decline, the reported incidence of neurocognitive decline after cardiac surgery varied extensively. The outcome suggests that 50–70% of patients experience cognitive decline when tested within one week of surgery, falling to 30–50% after 8–10 weeks, recovering to 10–20% at 1 year, and then declines again at 3–5 years. Currently, there is no widely accepted clinical definition of cognitive decline; therefore, it is possible that arbitrary definitions of decline have resulted in an overestimation of the incidence of decline. At present, there is no evidence to suggest that the long-term incidence of cognitive decline differs from that of nonoperative controls. Estimating long-term cognitive decline can be difficult, as normal ageing and dementia interfere with studies with older populations.

Further research is required to develop a more dynamic and nuanced picture of interactions between underlying pre- and perioperative risk factors. It is apparent that studies investigating isolated perioperative factors are insufficient to explain complex interactions between temperature, cerebral autoregulation, oxygen saturation, and brain metabolism. To date, isolated interventions and neuroprotective drugs aimed at improving cognitive outcome have proved to be largely ineffective. Literature examining underlying and perioperative risk factors associated with the pathogenesis of cognitive decline suggests that there is no single causative factor responsible for POCD. It seems likely that the causes are multifactorial, due to emboli, impaired perfusion, chronic cardiovascular disease, and inflammatory responses.

Interpreting the risk factors associated with postoperative cognitive decline, it seems that efforts to protect the brain during surgery are intrinsically linked with the need to control the progression of cardiovascular disease, especially in older patients. It is possible that patients may be exceeding a “threshold” of preexisting vulnerability where the brain's ability to compensate for injuries or inflammation during surgery is absent. It is also important to address that cardiac surgery equipment advances are a confounder within this review and should be considered. In summary, the literature examining underlying risk factors and perioperative risk factors associated with the pathogenesis of cognitive decline suggests that there is no single factor responsible for postoperative cognitive decline or single intervention capable of protecting the brain during surgery. Overall, the pathogenesis of cognitive decline following surgery still remains unclear.

Several factors have been associated with brain injury including hypoperfusion, arrhythmias, rapid rewarming, and inflammation (local or global) [34–36]. The process of preventing such brain injury involves prevention of such events occurring; however, to date no single intervention has successfully prevented cognitive decline, signalling an increased likelihood of a multifactorial aetiology. The advent of new technologies to prevent physiological stress on the brain has focussed on neuroprotective agents or perfusionist strategies. Although prevention has an important role, it would be ideal to develop methods of protecting or restoring neurocognitive decline to nearer preoperative baselines.

Cardiac surgery is a triumph of modern day medicine, and its acceptance as a safe procedure is widespread. Unfortunately, postoperative cognitive issues remain a consideration. As cardiac surgery procedures are now being challenged by less invasive methods, perhaps intraoperative transcranial Doppler monitoring, neuropsychological tests, and neuroimaging will play an increasingly important role in optimising treatment.

## Supplementary Material

To help with the commentary of the systematic review and ensure the transparent and complete reporting, a PRISMA chart has been attached to view the full selection process of the studies included. The diagram depicts the flow of information through different phases of the systematic review, mapping out the number of records identified, included and excluded.

## Figures and Tables

**Table 1:** Studies comparing cognition after cardiac surgery following administration of different types of anaesthetic.

Study	Study design	Number of patients	Type of anaesthesia/drug	Time of assessment	Outcome
Dumas et al., 1999 [37]	RCT	48	Fentanyl and early extubation	8 weeks	Improved cognition
Dowd et al., 2001 [38]	RCT	78	Propofol and lorazepam	6–12 months	Improved cognition
Bottio et al., 2007 [39]	Obsv.	50	Epidural anaes.	6 months	Improved cognition
Delphin et al., 2007 [40]	Obsv.	91	Sevoflurane and isoflurane	2 hours and 1 day	Improved cognition
Kanbak et al., 2007 [41]	RCT	40	Isoflurane, sevoflurane, and desflurane	3 and 6 days	Improved cognition
Hudetz et al., 2009 [42]	Obsv.	78	Ketamine	1 week	Improved cognition
Schoen et al., 2011 [43]	RCT	117	Sevoflurane and propofol	2, 4, and 6 days	Improved cognition
Kanbak et al., 2007 [41]	RCT	40	Sevoflurane and desflurane	3 and 6 days	Decline
Kadoi et al., 2003 [44]	RCT	180	Propofol and fentanyl	6 months	No difference
Silbert et al., 2006 [45]	Obsv.	300	Fentanyl	1 week, 3 months, 1 year	No difference
Kadoi and Goto, 2007 [46]	Obsv.	109	Sevoflurane	6 months	No difference
Lehmann et al., 2007 [47]	RCT	66	Sufentanil and midazolam	Discharge	No difference
Evered et al., 2011 [48]	Obsv.	281	General anaesthetics	1 week and 3 months	No difference
Parra et al., 2011 [49]	Obsv.	48	Sevoflurane	3 months	No difference
Royse et al., 2011 [50]	RCT	180	Desflurane andpropofol	Discharge and 3 months	No difference

Obsv.: observational.

**Table 2:** Studies investigating POCD associated with intraoperative blood pressure variation.

Study	Study design	Number of patients	Type of intervention	Time of assessment	Outcome
Gold et al., 1995 [8]	RCT	248	High (80–100 mmHg) versus low (50–60 mmHg) BP	6 months	Decline with lower BP
Siepe et al., 2011 [9]	RCT	92	High (80–90 mmHg) versus low (60–70 mmHg) BP	2 days	Decline with lower BP
Gottesman et al., 2007 [51]	Obsv.	15	Low MAP (50–70 mmHg)	3–5 days and 1 month	Decline with lower BP
Newman et al., 1995 [52]	Obsv.	237	Low MAP (50–60 mmHg)	Discharge	Decline with lower BP
Charlson et al., 2007 [10]	RCT	412	High MAP (57–90 mmHg) versus custom (capped at 90 mmHg)	6 months	No difference in outcome

Obsv.: observational.

**Table 3:** Studies investigating cerebral autoregulation during cardiac surgery in conjunction with neurocognitive tests.

Study	Study design	Number of patients	Cerebral autoregulation measures	Time of assessment	Outcome
Patel et al., 1993 [53]	RCT	70	Xenon-133 isotope clearance, CMRO_2_ (cerebral metabolic rate for oxygen), CERO_2_ (cerebral extraction ratio for oxygen)	6 weeks	Decline with impaired CA
Patel et al., 1996 [54]	RCT	70	CBF, CBFv, and O_2_ saturation were measured during 4 phases of surgery	6 weeks	Decline with impaired CA
Govier et al., 1984 [55]	Obsv.	67	Partial pressure of arterial carbon dioxide (PaCO_2_), clearance of xenon-133	Discharge	No difference
Newman et al., 1994 [12]	Obsv.	215	Xenon-133 clearance, CMRO_2_, cerebral AV difference (C[AV]O_2_)	Discharge	No difference

Obsv.: observational.

**Table 4:** Studies investigating whether biomarkers associated with inflammation and/or interventions aimed at reducing inflammation are associated with changes in cognition after surgery.

Study	Study design	Number of patients	Marker for cerebral damage	Time of assessment	Outcome
Fitch et al., 1999 [20]	RCT	35	Inhibition of complement activation by specific antibody and no antibody	Discharge	Improved cognition
Heyer et al., 2002 [21]	RCT	99	Inhibition of complement activation by heparin-coated CPB	5 days and 6 weeks	Improved cognition
Baufreton et al., 2005 [19]	RCT	30	Inhibition of complement activation by heparin-coated CPB	Discharge	Improved cognition
Skrabal et al., 2006 [22]	RCT	39	PMEA-coated circuits and noncoated circuits	7–10 days	Improved cognition
Wimmer-Greinecker et al., 1998 [56]	Obsv.	76	>S-100 and NSE	5 days and 2 months	Decline
Jönsson et al., 1999 [57]	Obsv.	132	>S-100	2 weeks and 2 months	Decline
Kilminster et al., 1999 [58]	Obsv.	130	>S-100	6–8 weeks	Decline
Rasmussen et al., 1999 [59]	Obsv.	35	>NSE	Discharge and 3 months	Decline
Derkach et al., 2000 [60]	RCT	27	>S-100 and NSE (deep and mild hypothermic)	6 months	Decline
Diegeler et al., 2000 [61]	RCT	40	>S-100 (on- and off-pump)	1 week	Decline
Georgiadis et al., 2000 [62]	Obsv.	190	>S-100	Discharge	Decline
Lloyd et al., 2000 [63]	RCT	125	>S-100 (on- and off-pump)	3 months	Decline
Basile et al., 2001 [64]	Obsv.	16	>S-100 and NSE	6 months	Decline
Rasmussen et al., 2002 [65]	Obsv.	15	>NSE	Discharge and 3 months	Decline
Farsak et al., 2003 [66]	Obsv.	50	>S-100	Discharge	Decline
Mathew et al., 2003 [67]	Obsv.	460	Reduced preoperative endotoxin immunity	6 weeks	Decline
Jönsson et al., 2004 [68]	Obsv.	56	>S-100	6 months	Decline
Kofke et al., 2004 [69]	Obsv.	28	Apo epsilon 4 allele, >S-100	8 and 24 hrs	Decline
Snyder-Ramos et al., 2004 [70]	Obsv.	64	>S-100 and NSE	Throughout 7 days	Decline
Kálmán et al., 2006 [71]	Obsv.	14	>Cytokine interleukin-6	1 week and 6 months	Decline
Ramlawi et al., 2006 [72]	Obsv.	42	>C-reactive protein	6 hours and 4 days	Decline
Lazibat et al., 2012 [24]	Obsv.	62	>S-100	2 days	Decline
Bayram et al., 2013 [25]	Obsv.	64	>S-100	1 week	Decline
Westaby et al., 2001 [26]	Obsv.	1001	>S-100 and NSE	5 days and 3 months	No difference
Mathew et al., 2005 [73]	Obsv.	440	Statin treatment	6 weeks	No difference
Plaschke et al., 2013 [74]	Obsv.	151	Preoperative serum anticholinergic activity	3 months	No difference

NSE: neuron-specific enolase, PMEA: poly-2-methoxyethylacrylate, and Obsv.: observational.

**Table 5:** RCTs investigating the efficacy of neuroprotection, or neuroprotective agents, in reducing cognitive decline after cardiac surgery.

Study	Number of patients	Type of neuroprotective drug	Time of assessment	Outcome
Grieco et al., 1996 [75]	29	GM-100 (ganglioside) or placebo	1 week and 6 months	Improved cognition
Arrowsmith et al., 1998 [76]	171	Remacemide or placebo	2 months	Improved cognition
Svensson et al., 2002 [77]	403	Mannitol, thiopental, MgSO_4_, lidocaine	2-3 weeks	Improved cognition
Wang et al., 2002 [29]	118	Lidocaine or placebo	9 days	Improved cognition
Uebelhack et al., 2003 [78]	64	Piracetam or placebo	3 days	Improved cognition
Szalma et al., 2006 [79]	98	Piracetam or placebo	6 weeks	Improved cognition
Haljan et al., 2009 [80]	32	Erythropoietin or placebo	Discharge and 2 months	Improved cognition
Hudetz et al., 2009 [42]	52	Ketamine or placebo	1 week	Improved cognition
Zhang et al., 2011 [81]	200	Benzyl alcohols or saline (placebo)	Discharge and 3 months	Improved cognition
Kong et al., 2002 [82]	245	Chlormethiazole/administration or placebo	4–7 weeks	No difference
Taggart et al., 2003 [83]	150	Imidazoles: low dose (10 mg) or high dose (100 mg) or placebo	5 days and 3 months	No difference
Mathew et al., 2004 [84]	914	Pexelizumab bolus, bolus plus infusion, or placebo	4 days and 1 month	No difference
Mathew et al., 2005 [73]	440	Hydroxymethylglutaryl-CoA reductase inhibitors	6 weeks	No difference
Hogue et al., 2007 [85]	174	17-beta estradiol or placebo	4–6 weeks	No difference
Mathew et al., 2009 [31]	241	Lidocaine or placebo	6 weeks and 1 year	No difference
Mitchell et al., 2009 [32]	158	Lidocaine or placebo	10 weeks and 25 weeks	No difference
Holinski et al., 2011 [86]	88	Piracetam or placebo	3 days	No difference

**Table 6:** Studies investigating POCD associated with temperature during cardiac surgery.

Study	Study design	Number of patients	Mean temperature (Celsius)	Time of assessment	Outcome
Grimm et al., 2000 [87]	RCT	144	(1) Normothermia: 37°C (2) Hypothermia: 32°C	1 week and 4 months	Improved cognition (with normothermia)

Shaaban-Ali et al., 2002 [88]	RCT	60	(1) Normothermia: 34°C (2) Hypothermia: 28°C	5 days	Improved cognition (with normothermia)

Nathan et al., 1995 [89]	Obsv.	30	Maintain ≤ 34°C	1 week	Improved cognition (with hypothermia)

Grocott et al., 2002 [90]	Obsv.	300	Post-op hypothermia only	6 weeks	Improved cognition (with hypothermia)

Kadoi et al., 2004 [91]	RCT	60	(1) Normothermia: 37°C (2) Hypothermia: 32°C	1 month	Improved cognition (with hypothermia)

Boodhwani et al., 2006 [92]	RCT	448	(1) Normothermia: 37°C (2) Hypothermia: 34°C	1 week	Improved cognition (with hypothermia)

Hiraoka et al., 2012 [93]	Obsv.	11	Hypothermia: 20–22°C	3 weeks and 6 months	Improved cognition (with hypothermia)

McLean et al., 1994 [94]	RCT	155	(1) Hyperthermia: >34°C (2) Hypothermia: <28°C	5 days and 3 months	No difference

Regragui et al., 1996 [95]	RCT	97	(1) Normothermia: 37°C (2) Hypothermia: 28°C & 32°C	6 weeks	No difference

Heyer et al., 1997 [96]	RCT	99	(1) Normothermia: 34°C (2) Hypothermia: 28°C	Discharge and 6 weeks	No difference

Kneebone et al., 1998 [97]	Obsv.	50	(1) Normothermia: 37°C (2) Hypothermia: 30–32°C	1 week	No difference

Reich et al., 1999 [98]	Obsv.	149	(1) Deep hypothermia: 12–15°C (<25 mins) (2) Deep hypothermia: 12–15°C (>25 mins)	1 month	No difference

Kaukinen et al., 2000 [99]	RCT	36	(1) Normothermia: 36-37°C (2) Hypothermia: 28°C	5 days and 11–23 months	No difference

Górna et al., 2001 [100]	Obsv.	33	No full text	3–10 days	No difference

Grigore et al., 2001 [101]	RCT	300	(1) Normothermia: 35.5–36.5°C (2) Hypothermia: 28–30°C	6 weeks	No difference

Kaukuntla et al., 2004 [102]	Obsv.	60	(1) Normothermia: 35°C (2) Differential temperature management	1 and 8 weeks	No difference

Reich et al., 2004 [103]	Obsv.	61	Monitoring during deep hypothermic arrest (28°C)	Discharge	No difference

Boodhwani et al., 2007 [33]	RCT	268	(1) Normothermia: 37°C (2) Hypothermia: 34°C	Discharge and 3 months	No difference

Kunihara et al., 2007 [104]	Obsv.	26	(1) Normothermia: 34°C (2) Hypothermia: 22°C	1 week	No difference

Obsv.: observational.

**Table 7:** Studies investigating POCD associated with the rate of rewarming during cardiac surgery.

Study	Study design	Number of patients	Mean temperature (Celsius)	Time of assessment	Outcome
Mora et al., 1996 [105]	RCT	138	(1) Rewarm 1-2°C (per increase) (2) Rewarm 3–5°C (per increase)	1–3 days, 7–10 days, and 1 month	Improved cognition with slower rewarm

Nathan et al., 2001 [106]	Obsv.	294	(1) Rewarm to 34°C (1°C per increase) (2) Rewarm to 37°C (3°C per increase)	1 week and 3 months	Improved cognition with slower rewarm

Grigore et al., 2002 [107]	Obsv.	100	(1) Rewarm to 32°C (max within 3 mins) (2) Rewarm to 37°C (max within 3 mins)	6 weeks	Improved cognition with slower rewarm

Kawahara et al., 2003 [108]	RCT	100	(1) Rewarm 1-2°C (per increase) (2) Rewarm 4-5°C (per increase)	1 month	Improved cognition with slower rewarm

Nathan et al., 2007 [109]	RCT	223	(1) Rewarm to 34°C (1°C per increase) (2) Rewarm to 37°C (3°C per increase)	1 week	Improved cognition with slower rewarm

Sahu et al., 2009 [110]	RCT	80	(1) Rewarm 1–3°C (per increase) (2) Rewarm 3–5°C (per increase)	5 days	Improved cognition with slower rewarm

Obsv.: observational.
