# Supplementary material for: Risk Factors Associated with Cognitive Decline after Cardiac Surgery: A Systematic Review
Source: Cardiovasc Psychiatry Neurol. 2015 Sep 30;2015:370612. doi: 10.1155/2015/370612 (PMC4605208; doi:10.1155/2015/370612)
Supplement: Supplementary file 1 — To help with the commentary of the systematic review and ensure the transparent and complete reporting, a PRISMA chart has been attached to view the full selection process of the studies included. The diagram depicts the flow of information through different phases of the systematic review, mapping out the number of records identified, included and excluded. [file 370612.f1.pdf]

Supplementary Material

PRISMA 2009 Flow Diagram

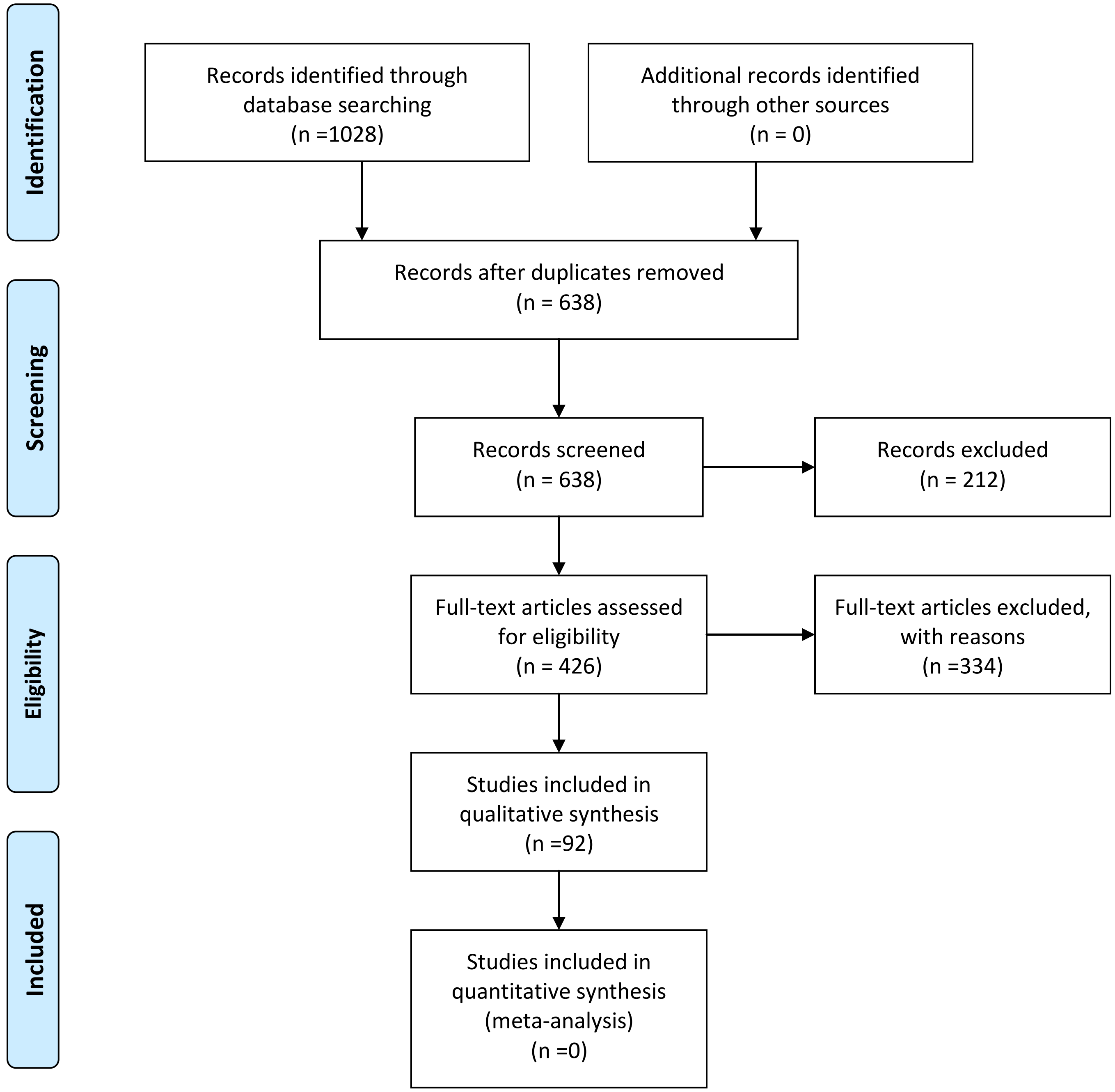

From: Moher D, Liberati A, Tetzlaff J, Altman DG, The PRISMA Group (2009). *P*referred *R*eporting *I*tems for *S*ystematic Reviews and *M*eta-*A*nalyses: The PRISMA Statement. PLoS Med 6(6): e1000097. doi:10.1371/journal.pmed1000097

For more information, visit www.prisma-statement.org.
